# What matters in end-of-life communication with nursing staff: an interview study among older people and their family caregivers

**DOI:** 10.1186/s12912-025-03882-4

**Published:** 2025-09-26

**Authors:** Fran B. A. L. Peerboom, Jolanda H. H. M. Friesen-Storms, Jenny T. van der Steen, Daisy J. A. Janssen, Judith M. M. Meijers

**Affiliations:** 1 Zuyderland Medical Center, Dr. H. van der Hoffplein 1, Sittard-Geleen, 6162 BG The Netherlands; 2 Department of Health Services Research, Care and Public Health Research Institute, Maastricht University, Duboisdomein 30, Maastricht, 6229 GT The Netherlands; 3 Research Center for Autonomy and Participation for Persons with a Chronic Illness and Academy for Nursing, Zuyd Health, Zuyd University of Applied Sciences, Nieuw Eyckholt 300, Heerlen, 6419 DJ The Netherlands; 4 Department of Public Health and Primary Care (PHEG), Leiden University Medical Center, Leiden, the Netherlands; 5 Radboudumc Alzheimer Center, Department of Primary and Community Care, Radboud University Medical Center, Nijmegen, The Netherlands; 6 Cicely Saunders Institute, King’s College London, London, UK; 7 Living Lab in Ageing and Long-Term Care, Maastricht University, Duboisdomein 30, Maastricht, 6229 GT The Netherlands; 8 Department of Expertise and Treatment, Proteion, Heythuyserweg 21, Horn, 6085 NH The Netherlands; 9 Department of Family Medicine, Care and Public Health Research Institute, Faculty of Health Medicine and Life Sciences, Maastricht University, Maastricht, The Netherlands; 10 Research Center for Nursing Leadership in a Changing Context and Research Center for Community Care, Academy of Nursing, Zuyd University of Applied Sciences, Nieuw Eyckholt 300, Heerlen, 6419 DJ The Netherlands

**Keywords:** Advance care planning, End-of-life communication, Family caregivers, Interview study, Nursing staff, Older people

## Abstract

**Background:**

End-of-life communication as part of advance care planning (ACP) aims to support older people to reflect on their values, needs, and wishes regarding the end of life. Previous studies have provided an understanding of the important aspects (“fundamentals”) of end-of-life conversations with older people from the perspective of nursing staff. Developing further understanding of the experiences and needs of older people and their family caregivers will help identify gaps in communication, guide nursing staff in providing successful ACP, and ultimately enhance person-centered care. We aimed to explore the experiences of older people and their family caregivers in home, nursing home and hospital settings regarding the fundamentals of end-of-life communication as part of ACP by nursing staff.

**Methods:**

Semi-structured interviews were performed with older people and their family caregivers about their experiences, opinions, and preferences before, during, and after recent formal end-of-life conversations. Data were analyzed thematically.

**Results:**

Eight older people and four of their family caregivers participated in three dyadic and six individual interviews between June 2023 and May 2024. Overall, participants felt it was difficult to describe and evaluate their experiences with the end-of-life conversations because they initially had no specific expectations about end-of-life conversations and approached them with an open mind. Three overall themes were composed comprising 11 fundamentals of end-of-life communication: “Navigating conversational phases: probing and reflecting” (e.g., readiness), “Fostering recognition and relational safety: acknowledging the older person” (e.g., feeling at ease, feeling seen while nursing staff attune to the older person, feeling a human connection), and “Engaging with family caregivers: valuing their role and well-being” (e.g., considering their well-being).

**Conclusion:**

Older people and their family caregivers prioritize feeling comfortable in natural and humane end-of-life conversations. They want to be seen, heard, and acknowledged as individuals with backgrounds, values, and needs, not just as patients with a disease. Nursing staff should be aware of the expectations of an older person and family caregiver in end-of-life communication and adjust their approach accordingly. The results of this study can help in developing effective strategies to ensure that end-of-life communication is tailored to the unique needs of older people and their family caregivers, fostering an informed approach.

**Supplementary Information:**

The online version contains supplementary material available at 10.1186/s12912-025-03882-4.

## Background

End-of-life (EOL) communication as part of advance care planning [1] (hereafter called EOL communication) includes proactive informal (i.e., spontaneous) and formal (i.e., planned) conversations between a person, a family caregiver, and a healthcare professional about future EOL care, the transition to the EOL phase, and death and dying from a holistic perspective [2–4]. Informal conversations about the EOL generally focus on exploring and identifying needs; these conversations may alternate with formal conversations, which focus on weighing options and making decisions [5]. ACP interventions may improve patient outcomes and empower patients by providing peace of mind and satisfaction in knowing their wishes are understood and respected. They may also offer a meaningful way to express love, trust, and reassurance to family caregivers, strengthening emotional connections, while helping patients gain greater clarity and control over future healthcare decisions, thereby preserving their autonomy [6, 7]. This communication process is particularly relevant for older people as they are often faced with the need to make (acute) decisions about (life-sustaining) treatments and EOL care due to the development of illness and cognitive and physical limitations [8, 9]. Timely involvement enables them to take a proactive role in decision-making about possible future EOL care [10].

Nursing staff (i.e., care assistants, certified nursing assistants, licensed vocational nurses, registered nurses, clinical nurse specialists, nurse practitioners, or general practice nurses) in home care, nursing home and hospital settings are well-positioned to play a central role in EOL communication, particularly due to the holistic nature of their care and the trust they often build with older people and their family caregivers [11]. These strong relationships between nursing staff and older people and their family caregivers can support meaningful EOL conversations [12]. Nursing staff have crucial roles in EOL communication as assessors, initiators, information providers, communicators, facilitators, actors, advocates, brokers, supporters, educators and managers [13]. Nursing staff could assess patients’ needs and preparations for discussion of ACP, identify the patients’ values and wishes for EOL care during admission and routine care, and, based on patients’ wishes, they refer patients to other professionals when appropriate. Nursing staff also act as mediators between patients and their families, and between patients and their health care teams [13–15].

Despite their crucial role, nursing staff often feel uncomfortable in discussing death, lack adequate training and guidance for engaging in EOL communication, and are uncertain about appropriate timing, roles, and responsibilities [16]. As such, EOL communication is widely recognized as a complex and advanced skill that requires training and support. Insight into the “fundamentals” (most important aspects) of holistic and person-centered EOL communication is needed to help nursing staff guide EOL conversations, provide future education and training, and overcome barriers [17]. We define fundamentals as the important aspects of EOL communication such as the prerequisites, competencies, activities, and values involved in preparing for, carrying out, and evaluating EOL communication.

This study is part of a larger project called LISTEN (Learning about the essentIal fundamentalS of EOL communicaTion in palliativE Nursing care; Box 1), in which we aim to build a theoretical framework of the fundamentals of EOL communication performed by nursing staff with older people. As part of this project, we previously conducted a scoping review to explore and identify the fundamentals of EOL communication [18], followed by an interview study among nursing staff [19]. Other studies have described fundamentals of EOL communication performed by healthcare professionals from the perspectives of patients in general and their family caregivers. These fundamentals include, for example, trust in healthcare professionals [20], establishing a meaningful connection [21], recognizing and reflecting on emotions [21], and being remembered [22]. More knowledge is still needed regarding the fundamentals of EOL communication provided by nursing staff from the perspectives of older people and their family caregivers across different settings. Understanding their experiences and needs will help identify fundamentals of EOL communication, guide nursing staff in providing successful ACP, and ultimately enhance person-centered care. This is crucial for developing effective strategies to ensure that EOL communication is tailored to the unique needs of older people and their family caregivers, fostering an informed approach. Therefore, the present study aimed to explore the experiences of older people and their family caregivers in home, nursing home and hospital settings regarding the fundamentals of EOL communication as part of ACP by nursing staff.

**Table 1:** Previous findings in the LISTEN project that informed the current study

Our previous **scoping review** [18] showed that nursing staff attune EOL communication to the values and needs of older people to approach the process in a person-centered manner. This approach requires additional fundamentals, such as building a relationship, assessing readiness, timing and methods to start the conversation, attention to family relationships, and listening and non-verbal observation skills. Building a trusting relationship especially helps nursing staff attune EOL communication to the values and needs of older people to approach the communication process in a person-centered manner [18]. The scoping review included only two studies describing the perspectives of older people and their family caregivers and focused mainly on the hospital setting [18]. Our previous **interview study** with nursing staff [19] across different settings described additional fundamentals such as the ability to communicate about the EOL, learning by doing, and using intuition, providing a rich understanding of the fundamentals from the perspective of nursing staff. The study emphasized the importance of moving along with the older person, connecting, adapting, and relinquishing control over the outcome of the conversation whenever possible.	

## Design

This is a qualitative descriptive study in which semi-structured interviews with older people and their family caregivers were conducted to investigate fundamentals of successful EOL conversations [12, 23]. Older people and their family caregivers were asked to reflect on and describe their experiences with their most recent EOL conversation with nursing staff. We used the findings of our previous scoping review and interview study with nursing staff (Box 1) to inform this study.

This study was reported in accordance with the Consolidated Criteria for Reporting Qualitative Research [24].

## Materials and methods

### Population and domain

Older people (> 65 years old) and their family caregivers were included using convenience sampling, with the intention of capturing variation across characteristics such as age, gender, ethnicity, educational level, and healthcare setting. However, given the small sample size, achieving full heterogeneity was not feasible, and the final sample reflects limited diversity (further explained in the Results section).

Older people were invited to participate if they recently had at least one EOL conversation with nursing staff (i.e., care assistant, certified nursing assistant, licensed vocational nurse, registered nurse, clinical nurse specialist, nurse practitioner, or general practice nurse) when receiving home care, nursing home care, hospital care (inpatient or outpatient care), or support from a general practitioner office. In the Netherlands, care assistants and certified nursing assistants are allowed to engage in end-of-life conversations, which may differ from regulations in other countries. Conversations that occurred within the 8 weeks prior to the interview were included to ensure participants could recall the EOL communication in sufficient detail. Conversations were part of regular healthcare and were not manipulated as part of the study. Family caregivers of the older people were invited to participate if they had been present during at least one of the EOL conversations with the older person.

### Working group and consultants

To support the study, the research group consulted an interprofessional working group (*n* = 16), which is also part of the larger LISTEN project. This group comprised a patient representative; nursing staff with different educational levels working in hospital, home, and nursing home settings; members of a transmural palliative care consultation team; a spiritual caregiver; and other experts in palliative care, geriatric nursing care, and nursing education. The working group was consulted on three occasions to discuss the content of the interview guide, selection of participants, analysis approach, and interpretation of the results. In addition to the working group, a smaller group of consultants (two patient representatives and two experts in ACP, palliative care, and qualitative research) was consulted once to discuss the content of the interview guide.

### Recruitment

Older people and their family caregivers were recruited by three healthcare organizations providing care in multiple healthcare settings in the south of the Netherlands. The LISTEN project has partnered with these organizations. Participants were recruited from June 2023 to May 2024. After ongoing review and refinement of the recruitment process by the research team due to recruitment difficulties, the palliative care consultation teams at the healthcare organizations, nursing staff in the working group, and nursing staff within the research team’s professional network assisted in recruiting participants. Nursing staff approached older people and their family caregivers first and informed them about the study using a recruitment letter. If they expressed interest in participating, FP (a female nurse scientist and Ph.D. candidate) contacted them via telephone to start the informed consent procedure and schedule an interview.

### Procedures

The interdisciplinary research team consisted of researchers experienced in qualitative research, interviewing about sensitive topics, and ACP. Semi-structured interviews were performed face-to-face at locations of participants’ choice. Before the interviews, participants were asked to sign the informed consent form, and baseline characteristics (i.e., gender, age, and healthcare setting) were collected verbally by FP. Based on the preferences of participants, the interviews were conducted either individually or in pairs by FP. The interviews were audio-recorded. Field notes were recorded during and after each interview.

### Interview guide

The fundamentals defined within the described themes from the results of the scoping review (e.g., preparing for EOL communication and having a professional attitude) [18] were used as input for the first draft of the interview guide. The research group, working group, and project consultants discussed the first draft; thereafter, the interview guide (Appendix A) was established. The guide included questions about experiences, opinions, and preferences regarding fundamentals before, during, and after EOL conversations. Prompts were provided in addition to each interview question, including “Could you please explain…” or “How did you experience…” FP conducted two pilot interviews to become familiar with the interview guide and test its applicability. The interview guide was considered applicable. The data from the pilot interviews were not used in the analysis because the participants did not meet the inclusion criteria (i.e., age of < 65 years and EOL conversation more than 8 weeks ago).

After every one or two interviews, transcripts were analyzed, and the interview approach and content were adjusted when necessary, emphasizing certain questions in the interview guide to allow for a deeper exploration and understanding of the research topic (e.g., delving more into how specific fundamentals were noticed and experienced by older people and their family caregivers).

Because the analysis of our previous interview study among nursing staff [19] was conducted in part in parallel with the present study, we were also able to delve deeper and explore the perspectives of older people and their family caregivers about emerging conflicting findings. For example, we asked whether it is appropriate for nursing staff to show emotions and how well-prepared they need to be.

### Data analysis

**Fig. 1:**
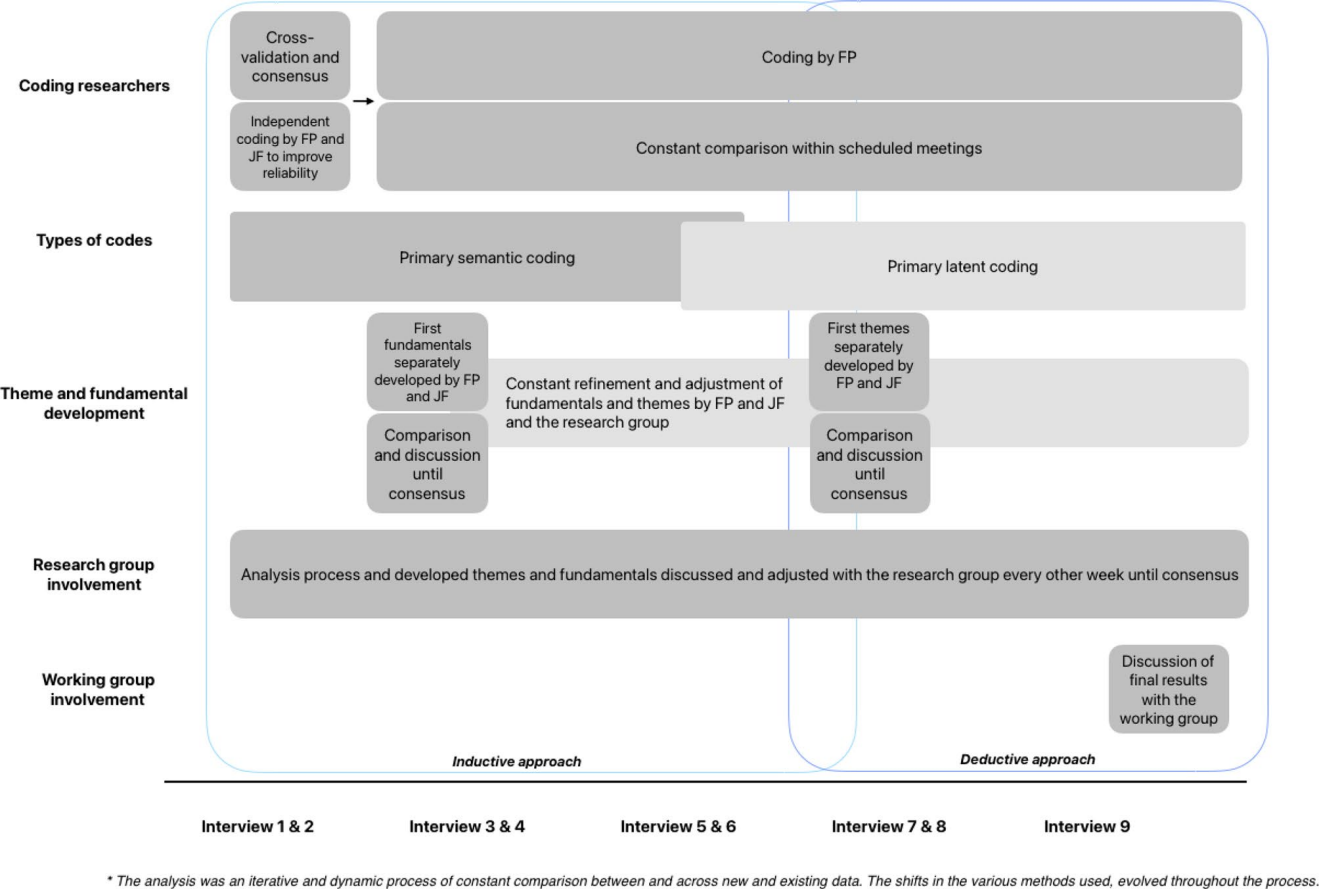
Components of the analytic process*

The audio-recordings were transcribed verbatim and analyzed using the reflexive thematic analysis approach of Braun and Clarke [25]. Transcripts and preliminary findings were not returned to participants. The analysis was conducted from a constructionist epistemology perspective with an experiential orientation [26]. We considered what participants said in the interviews to be implicit regarding meaning and experience, which required interpretation and reading between the lines to eventually recognize themes and fundamentals. In addition, the view of participants regarding the importance of specific fundamentals guided the development and interpretation of codes and themes. Therefore, we further explored the meaning that participants ascribed to the fundamentals. A schematic overview of the analysis process is displayed in Fig. 1. During this process, the research group remained close to the terminology used by participants. Data collection ended when no new themes appeared to emerge in relation to the study objectives, suggesting data saturation [27, 28]. Saturation was cross-checked and discussed after every three to four interviews with a second researcher (JF). Any potential biases or assumptions the researchers made during data collection and data analysis that were relevant to the description of the fundamentals, such as “there were only reflections on formal EOL conversations” or “this interview showed similar findings to the previous interview regarding lack of expectations,” were noted in memos and reflected on for rigor and reflexivity purposes. ATLAS.ti (version 24.1.1) was used to support the analysis.

### Ethical considerations

The study was conducted according to the principles of the Declaration of Helsinki and the Medical Research Involving Human Subjects Act [29]. It has been declared exempt by the Medical Research Ethics Committee of Zuyd University of Applied Sciences and Zuyderland Medical Center (April 12, 2024, registration number Z20230031).

## Results

### Participants and demographic data

After a complex recruitment process that required the involvement of many different healthcare professionals, a total of six individual interviews and three dyadic interviews were conducted from June 2023 to May 2024 at the participants’ homes. A total of eight older people (“O”) and four of their family caregivers (“FC”) participated in these interviews (Table 1). All participants were recruited in response to formal EOL conversations. Every referred eligible person agreed to participate.

The interviews had a median duration of 57 min (range: 39–82). Five female and three male older people participated, with a median age of 85 years (range: 70–96). Among them, two older people received care in a nursing home setting; three received care in a hospital; and three received support from a general practitioner office. Three female and one male family caregivers participated, with a median age of 74 years (range: 71–79). Each family caregiver was the older person’s partner. Two family caregivers of older people who received hospital care and two of those who received support from a general practitioner office were recruited. No family caregivers of older people who lived in a nursing home were recruited.

**Table 2:** Demographic characteristics

Participant ID	Male/female	Age category (years)	Setting	Main medical condition
**Older people**				
O1	Female	70–75	Hospital	Respiratory disease
O2	Female	96–100	Nursing home	Metabolic disease
O3	Male	70–75	Hospital	Cardiovascular disease, respiratory disease, hepatic disease
O4	Male	81–85	Hospital	Neurologic disease, cardiovascular disease
O5	Male	70–75	General practitioner office	Cancer
O6	Male	91–95	General practitioner office	Cancer, respiratory disease
O7	Female	91–95	General practitioner office	Renal disease, cardiovascular disease
O8	Male	86–90	Nursing home	Metabolic disease, neurologic disease
**Family caregivers**				
FC1	Female	70–75	Hospital	Cardiovascular disease, respiratory disease, hepatic disease*
FC2	Female	70–75	Hospital	Neurologic disease, cardiovascular disease*
FC3	Female	70–75	General practitioner office	Cancer*
FC4	Male	76–80	General practitioner office	Neurologic disease*

*Indicates the medical condition affecting the older person (i.e., the family caregiver’s partner), not the family caregiver themselves

### Findings

**Fig. 2:**
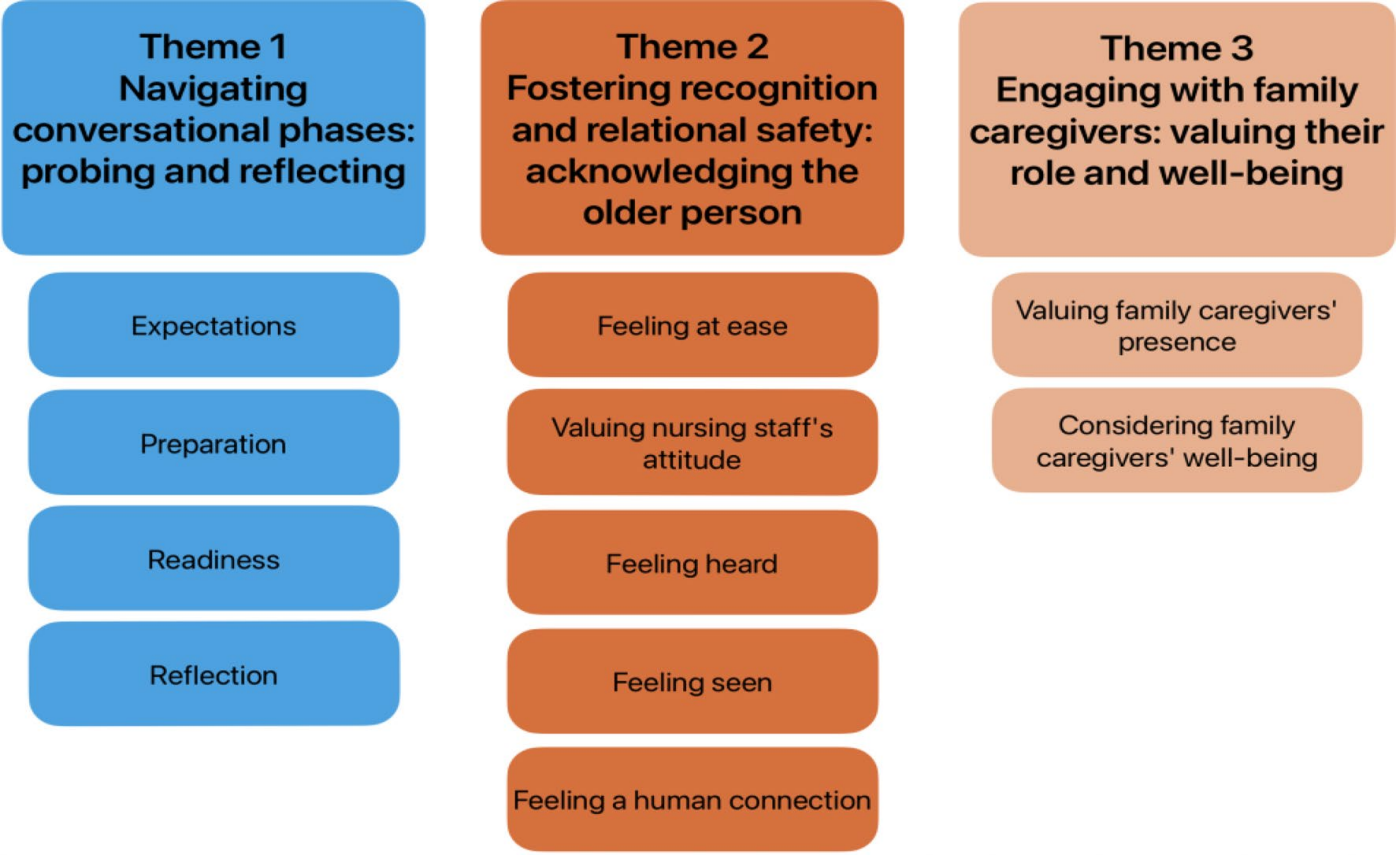
Themes and fundamentals

An overall finding was that the older people and family caregivers indicated that they initially had no specific expectations about EOL conversations and approached them with an open mind. By applying a reflexive thematic analysis approach, we recognized three overarching themes clustering 11 fundamentals based on the experience of the participating older people and their family caregivers (Fig. 2).

Saturation seemed to occur around the seventh or eighth interview. We interpret the apparent point of saturation with caution, given the small sample size and limited variation in participants’ perspectives, particularly regarding their expectations of EOL communication.

#### Theme 1: Navigating conversational phases: probing and reflecting

Although the older people and family caregivers did not explicitly describe conversational phases of EOL communication, they did suggest that it began with probing and concluded with reflection, thereby identifying certain phases.

##### Expectations

The older people and family caregivers had an open mind about EOL conversations and did not have specific ideas, expectations, and concerns about the content, structure, or fundamentals nursing staff apply.

*I just let it [the EOL conversation] come to me because I did not expect it to actually be that extensive. (O1*,* hospital)*

Most older people and family caregivers indicated that it was most important for them to be treated and seen as persons, feel valued, and perceive the conversation positively.

*I appreciate it when someone truly shows interest in us [older person and family caregiver]. That makes it personal. […] Then at least I feel like a real person. (O8*,* nursing home)*

##### Preparation

Due to their lack of expectations of EOL conversations, the older people and family caregivers usually made little to no preparation:

*We [older person and family caregiver] went in blank. I thought*,* ‘We will see*,* but it will not be fun*,*’ right? When she [nurse practitioner] says*,* ‘I want to talk to your wife and children*,*’ then you think ‘This must have a reason.’* (FC1, hospital)

Some older people and family caregivers indicated that they were unaware of the scope of the EOL conversation in advance. One older person and his family caregiver expressed a desire to have been informed in advance so that they could mentally prepare for the conversation. However, the other participants did not find any problem with being unaware of the scope of the conversation in advance. When the older people did prepare for the conversation, they did so by using an information leaflet they had previously received from nursing staff, completing a questionnaire they had received (e.g., ACP readiness questionnaire or a disease specific questionnaire), or writing down questions they wanted to ask beforehand. They indicated that they were satisfied with this approach, although it was not always followed:

*Yes*,* there were forms. I could fill them out. But*,* yes… whether I filled them in at all… I do not think so.* (O1, hospital)

*So we know that she [the nurse] is coming on April 5th. […] Now should there be anything in that period up to April 5th*,* we either remember it or we write it down*,* like*,* ‘We should ask that anyway [during the next EOL conversation].* (O5, general practitioner office)

##### Readiness

Although the older people and family caregivers made little to no preparation for the EOL conversation and had no specific expectations, they still indicated that being ready for such a conversation or for difficult topics relevant to EOL communication (e.g., death and dying) could contribute to a good and deep conversation. This was not a fundamental they were concerned about before the EOL conversation. However, they sometimes became aware of it during the conversation, afterward when reflecting on it, or only because the interviewer specifically asked about it. The older people indicated that nursing staff adapted to this readiness, for example, by carefully introducing difficult topics during the first conversation if the older person was not ready. Previous personal experiences with the EOL or with EOL communication (i.e., before becoming ill) could contribute to this readiness. For example, some older people and family caregivers explained that they had discussed EOL matters with each other or that they had previously experienced the death of a family member or friend. These experiences made EOL communication with nursing staff easier by preparing them for more serious topics, such as death and dying:

*The more often we [older person and family caregiver] talk about it [end of life*,* death]*,* the more likely [habitual] it [having these conversations] becomes for you [family caregiver]. And also for myself [older person]. I think it is going to be a little easier that way.* (O3, hospital)

The older people who had not previously engaged in other EOL conversations indicated that they would have liked to. This was because they realized that the earlier older people had this conversation, the earlier they started thinking about future care. Being or becoming aware of the importance of and reason for the conversation contributed to some older people’s readiness and willingness to engage in EOL communication.

##### Reflection

Most older people and family caregivers reported reflecting on an EOL conversation together afterward. In most cases, such a conversation prompted consideration of treatment wishes and their importance. However, in one case, reflection was not always considered necessary:

*There are also people who hardly ever talk about it [the end of life]. For them*,* it [the conversation] makes an impact. They have to think about [reflect on] it again. I do not have to think [reflect] at all; I just have a conversation*,* and then it is okay.* (O2, nursing home)

One older person and his family caregiver mentioned that they received a written summary of the EOL conversation afterward. This summary was seen as useful because it reminded them of the issues they had discussed and allowed them to reflect on and reconsider such issues.

Most older people and family caregivers indicated that they had follow-up conversations with nursing staff to reflect on or follow up on the EOL conversation. They valued this renewed contact because it made them feel seen and heard, as though they had not been forgotten.

#### Theme 2: Fostering recognition and relational safety: acknowledging the older person

Although older people and family caregivers repeatedly expressed during the interviews that they had difficulties articulating why they found an earlier EOL conversation good, they frequently emphasized that the most important aspect of this conversation was fostering recognition and relational safety through nursing staff acknowledging them. This was experienced as feeling comfortable, being seen and heard, and having a natural, humane conversation. Sometimes, these fundamentals were related to the attitudes of nursing staff, while at other times, they were linked to communication techniques.

##### Feeling at ease

The older people and family caregivers particularly appreciated EOL conversations that felt natural and relaxed and were open (e.g., open to discussing any topic). In this way, they felt safe and at ease and tended to share more information:

*They [nurses] must be very open. It [the EOL conversation] then feels so personal. It [the EOL conversation] feels just like home then. And I think that is very important.* (O7, general practitioner office)

Furthermore, the older people and family caregivers indicated that nursing staff should discuss sensitive topics in a lighthearted manner whenever possible and allow for laughter during the conversation. This would help create a more relaxed atmosphere and enable them to feel more at ease:

*Keep smiling. […] Then you also notice that it [the EOL conversation] feels pretty relaxed already.* (O4, hospital)

##### Valuing nursing staff’s attitude

The attitude of nursing staff played an important role in creating a space for open communication. For example, the older people and family caregivers emphasized the importance of nursing staff sitting down for the conversation in a relaxed manner and approaching the conversation in a personal and fluid way, rather than in a businesslike and distant manner:

*If such a person [the nurse] is relaxed*,* then I think the conversation also runs a little easier than with a tensed attitude. […] That is also how most things come to the table.* (FC3, general practitioner office)

Other attitudes of nursing staff the older people and family caregivers found important were kindness, trustworthiness, and empathy. Moreover, during the conversation, they valued nursing staff who were caring, non-interruptive, accepting, non-judgmental, understanding, and honest. These features fostered a trusting relationship between nursing staff and the older people, consequently encouraging the latter to be more open during the conversation:

*I think she [the nurse] empathizes very well. […] Because she understands. […] She understands your feelings*,* how you experience*,* you feel that she understands. And that gives me a very trusting impression.* (O2, nursing home)

*Even if it is painful. Just tell everything honestly [during an EOL conversation]. Then you know where you stand. […] That is how I prepare myself to be able to cope [with the rest of the conversation and the future].* (O7, general practitioner office)

##### Feeling heard

Nursing staff being aware of the current situation and previous conversations with the older people and family caregivers was considered valuable. This resulted in feeling heard and having a real conversation:

*Because she [the nurse] is interested in you*,* she remembers what you have*,* she remembers the little problems. Because it is really an art to know people well*,* to still remember what they actually have.* (O2, nursing home)

In addition, active listening during the conversation was considered important. The older people and family caregivers explained that they could immediately discern whether nursing staff were actively listening and thus being consciously present during the conversation. This was evidenced by nursing staff continuing to ask about what the older people and/or family caregivers were saying or the cues they were giving and returning to issues discussed earlier in the conversation or during another conversation. The older people and family caregivers indicated that this approach not only made them feel seen and heard but also demonstrated the importance of the conversation to nursing staff and its positive impact on the relationship:

*If she refers to an earlier conversation. […] Then it has been real. Then it has remained in her mind*,* it has gone a little deeper. Not like in one ear and out the other. I think that is very important*,* to become close to someone. Yeah. Yeah*,* I think that is really important.* (O7, general practitioner office)

*I quickly notice if someone is interested in what I say. I can tell very quickly. Some people listen but are not present. Others encourage me to go on and tell.* (O8, nursing home)

A potential challenge identified by the older people and family caregivers was asking too many questions, which could impede the flow of the conversation. Some older people and family caregivers suggested that allowing for silences could help address this. One older person explained that obtaining a substantial amount of information through few well-formulated questions is essential for a good EOL conversation. Furthermore, the older people and family caregivers indicated that the conversation and communication should be clear. For example, they felt it was important for nursing staff to speak calmly and be direct when necessary (e.g., when the older person does not understand the seriousness of something or when the older person specifically asks for it). Additionally, they suggested that nursing staff should provide further explanations when asked for clarification, such as by illustrating information on paper:

*She [the nurse practitioner] wanted to draw it [the course of the disease] for him. So that he could understand it better. Because he said many times that he did not understand it. […] So she would explain it over and over again.* (FC2, hospital)

##### Feeling seen

The ability of nursing staff to sense and use intuition was also recognized by the older people and family caregivers. For example, they noted the importance of sensing what a conversation should focus on or identifying when something is affecting the older people and family caregivers. This approach made the older people and family caregivers feel seen:

*She [the nurse] notices very quickly if something is wrong. (O7, general practitioner office)*

*Everyone [every nurse] is also different in this regard. I cannot say like*,* ‘You [nurse] should do this or that.’ You [the nurse] must be able to sense it a little bit.* (O1, hospital)

The older people and family caregivers suggested that nursing staff should also use their intuition to tailor the content of the conversation to their needs and preferences, taking into account any potential changes in their health condition that might occur over time.

*The conversations*,* they make sense. She [the nurse] gives answers to [responds to] the experiences you share or things you feel; she [the nurse] pays attention to that*,* and she [the nurse] feels that.* (O2, nursing home)

In addition, nursing staff should gently explore their values, wishes, and needs and slowly work toward more difficult topics, such as death and dying.

##### Feeling a human connection

Being able to see nursing staff as human beings, wherein they are authentic and occasionally share something personal, was considered essential to the conversation by the older people and family caregivers. Some older people indicated that the humanity of nursing staff contributes to a more open conversation, allowing them to express themselves, ensuring that the person behind the “patient” is acknowledged, and consequently fostering a trusting relationship:

*I think it is important to know something about the staff*,* reciprocally. […] It makes you feel at home*,* yes. It is like being with your children. […] It feels the way it should*,* right? The warmth. The warmth of two sides. […] Then we are the same*,* the uniqueness of them and me. So*,* open towards each other. That you know each other well. […] That also makes for a deeper conversation.* (O7, general practitioner office)

*I think it [a humane conversation] is also easiest for her [the nurse]; to see the person that way*,* in a normal conversation.* (O5, general practitioner office)

When specifically asked by the interviewer, the older people and family caregivers also indicated that showing emotions could be part of the humanity of nursing staff. However, this rarely happened:

*You do not expect that [to show emotion]. […] But when it happens*,* it happens. It is also human*,* of course. […] Because underneath everything they do*,* they remain a human being with their own personality. […] Respect everyone as they are. I think that is very important.* (FC3, general practitioner office)

Nursing staff were also seen as independent, approachable, and close to the older people and family caregivers. They would always be there for the older people, could speak the same language, had the older people’s best interests at heart, and were willing to talk about anything:

*You can talk about anything [with the nurse]. So that is pleasant. You can actually say*,* tell*,* whatever you want. […] And she [the nurse] also gives her answers to that. That is pleasant.* (FC3, general practitioner office)

The older people and family caregivers reported that nursing staff are often more accessible than physicians. They also perceived nursing staff as “umbrella professionals,” who are knowledgeable and often protective of older people in interdisciplinary teams:

*I trust her [the nurse practitioner] completely. I feel that she is really the overarching person over my life.* (O2, nursing home)

*You can talk to her [the nurse] a little more freely than you can talk to the doctor. (O6, general practice)*

#### Theme 3: Engaging with family caregivers: valuing their role and well-being

ACP was noted to affect not only the older people but also the family caregivers. The family caregivers and older people indicated that they felt it was important for both to be present during the conversation whenever possible. The older people particularly recognized the special role of family caregivers in complementing them during EOL conversations while also emphasizing the importance of considering the well-being of family caregivers.

##### Valuing family caregivers’ presence

The older people emphasized that the presence of their family caregivers during EOL conversations is essential. Family caregivers should be informed about and agree with what is being discussed, as they can complement the older person, ask clarifying questions during the conversation, and support reflection afterward:

*He [my son] listened to everything. And from time to time*,* he would ask a question*,* like*,* ‘These medicines*,* are they good for her [the older person]?’* (O1, hospital).

The family caregivers also felt that their own presence was essential to support the older person, agree on issues, and be informed but that they only added to the content of the conversation when necessary:

*Let him [the older person] tell*,* and then what needs to be added*,* I will add. […] You [the older person] tell your story; I will add to it if necessary. I can also say something. And that is nice anyway.* (FC3, general practitioner office)

##### Considering family caregivers’ well-being

The older people also found it crucial that their family caregivers are prepared to go on without them and can express their wishes and concerns about this during the conversation:

*She [the nurse] just walks inside our house. And my wife can also tell her story [her worries about moving on alone]. Yes*,* it is good for her to talk; yes*,* it is important. That is why we like to have these conversations together.* (O5, general practitioner office)

In addition, the older people and family caregivers felt that the well-being of family caregivers should also be considered during the conversation:

*When she [the nurse] comes over*,* she [the nurse] comes for the patient [the older person]*,* but she [the nurse] will also always come to me and ask*,* ‘How are you?’ They just know. […] That is also important*,* how the person next to them [the older person] deals with it.* (FC3, general practitioner office)

## Discussion

This study explored and described the experiences of older people and their family caregivers in home, nursing home and hospital settings regarding the fundamentals of EOL communication as part of ACP by nursing staff. Overall, the older people and family caregivers indicated that they had no real expectations regarding the content, approach, and process of EOL conversations and approached them with an open mind. They found it most important to feel comfortable in natural and humane EOL conversations, be seen and heard, and be treated holistically as people with backgrounds, values, and needs, not just as patients with a disease. The fundamentals of EOL communication we recognized were related to three overall themes: “Navigating conversational phases: probing and reflecting” (e.g., readiness), “Fostering recognition and relational safety: acknowledging the older person” (e.g., feeling at ease, feeling seen while nursing staff attune to the older person, feeling a human connection), and “Engaging with family caregivers: valuing their role and well-being” (e.g., considering their well-being).

The findings of this study, particularly older people’s and family caregivers’ emphasis on feeling seen, heard, and treated with humanity during EOL conversations, closely reflect the core humanistic values that underpin both palliative and nursing care. For example, these fundamentals align with the Humanistic Nursing Theory, which highlights the significance of intersubjective relationships, mutual presence, and the recognition of personhood [30, 31]. This theory advocates for a model of care grounded in authentic human connection, rather than one centered on task completion or procedural efficiency [30, 31]. Furthermore, our findings can be interpreted through the lens of the ethics of care, which place relational autonomy, attentiveness, and emotional responsiveness at the heart of ethical caregiving [32]. These principles are similarly reflected in person-centered care models, which emphasize holistic, individualized care that respects the values, needs, and lived experiences of the people involved [33, 34]. The findings in our study are consistent with these frameworks.

This shared orientation toward human connection and emotional attunement may help explain why older people and family caregivers in our study had few specific expectations regarding the content, approach, and process of EOL communication, a finding also supported by previous research [35, 36]. This became particularly evident in the following fundamentals: expectations, preparation, and readiness. The older people were open-minded about EOL conversations and did not know what to expect. Although they were not prepared for these conversations most of the time, some older people indicated that being ready for EOL communication could contribute to a good and deep conversation. This preparation could, for example, be supported by having a nurse explain what to expect, building on previous experiences with EOL communication (e.g., personally or with another family member or friend), or using a tool. Some older people and family caregivers also expressed a desire to have been informed of the scope of the EOL conversation to be able to prepare mentally. However, this was not something that concerned them before the EOL conversation; they became aware of it during the conversation or even afterward when reflecting on the conversation or because the interviewer specifically asked about it. These findings indicate that some older people may benefit from a more proactive approach from nursing staff, similar to other research [37–39].

Nursing staff can support a more proactive approach to EOL communication by timely initiating conversations and preparing an EOL conversation with older people and their family caregivers, either verbally or by providing tools such as a questionnaire or an information leaflet. According to other research, preparing older people or patients in general for EOL communication is considered a common first step in ACP. For example, the systematic review by Fahner et al. on interventions guiding ACP conversations revealed a framework of four phases: preparation, initiation, exploration, and action [40]. Other studies have also shown that older people demonstrate varying degrees of reticence, evasion, or reluctance to initiate EOL conversations, usually assuming that nursing staff will take the lead. However, nursing staff are often hesitant to initiate these discussions themselves [36]. The same may be true for preparing for EOL conversations, but this is not explicitly described in scientific literature. As expectations and preferences in EOL communication may differ, nursing staff should be aware of the preferences of older people and their family caregivers and adjust their approach accordingly.

Another example of the value of nursing staff being aware of the preferences of older people and their family caregivers can be seen by comparing the key finding of our previous scoping review with the findings of our previous and current interview studies. A key finding of our scoping review was that building a trusting relationship helps nursing staff align EOL communication with the values and needs of older people to approach the communication process in a person-centered manner [18]. This finding was also supported by our previous interview study among nursing staff [19]. Based on the present study findings, older people and their family caregivers agree but add that in building this trusting relationship, they especially value a human connection where nursing staff are authentic and occasionally share something personal (e.g., own emotions).

Our previous interview study among nursing staff also emphasized the importance of moving along with the older person, connecting, and adapting [19]. Many of the fundamentals noted in that study could be traced back to the basics of nursing and the humanity of a conversation [19]. In addition, our present study highlights the importance of relinquishing control over the content, structure, or outcome of EOL conversations. This person-centered and humane approach, valued by older people and their family caregivers, was also highlighted in our previous interview study. It makes older people and their family caregivers feel seen and heard, potentially because they may not be explicitly concerned about the content of the EOL conversation or the fundamentals used by nursing staff.

In contrast, in our previous interview study, nursing staff described in great detail how they engaged in EOL communication with older people, what competencies and communication techniques they needed, and what steps they took to achieve them [19]. This contrast suggests that fundamentals of a good EOL conversation depend on the perspective from which they are viewed. Although nursing staff often value an EOL conversation that has a certain structure or desired outcome and are concerned with addressing the appropriate topics from a nursing perspective (e.g., treatment preferences), older people and their family caregivers are more concerned with connecting and being seen and heard during ongoing, holistic, natural, and humane EOL conversations. Both approaches to EOL communication may be appropriate provided they are balanced with the needs of older people and their family caregivers.

Given the finding that older people and their family caregivers are primarily concerned with connecting and being seen and heard to engage in natural and human conversations, it may also suggest reflection on the value and appropriateness of conversation tools for nursing staff in EOL communication. As found in our previous interview study [19], these tools may be convenient for novice nursing staff who want to gain initial experience with EOL communication. They may help build confidence and skills, serve as a fallback when formal conversations do not go smoothly, and support reflection on such conversations. However, our studies also showed that relying too heavily on these tools could hinder openness, connection, and presence during EOL conversations based on the perspectives of nursing staff, older people, and family caregivers. These tools should not take the lead in EOL conversations to meet the preferences and needs of individuals engaging in EOL communication. Nursing staff need to be trained in the proper way of using such tools as an initial introduction and familiarization with EOL conversations. In addition, nursing staff must receive support through training, teaching, or coaching to take ownership of and remain consciously present during EOL conversations, applying the fundamentals identified in this study and our previous studies.

### Strengths and limitations

This study has several strengths and limitations. A limitation is that participants were selected from different settings, which compounds the complexity of the type of EOL conversation the participants may have experienced. A strength, however, is that these participants described common universal experiences across the settings from which general fundamentals to include in the theoretical framework of the LISTEN study could be derived. Another limitation is that a limited number of older people and their family caregivers were recruited in a relatively long and complex recruitment process in which nursing staff seemed to act as gatekeepers that could protect older people and their family caregivers from what they believed might overwhelm them [41]. This possible protective approach may have led to the exclusion of older people who were perceived as more vulnerable or emotionally burdened. As a result, the study might have missed perspectives from those who could have found these conversations more challenging. In addition, all participants were recruited by nursing staff in response to formal EOL conversations. The older people, family caregivers, and nursing staff were either often unaware or became aware late that an informal EOL conversation was taking place, which made both recruitment and reflection during the interviews difficult. The participants may represent a selected sample. A third limitation is that all family caregivers in this study were spouses of the older people, which limits the transferability of our findings to family caregivers in other types of relationships [42–44]. Spouses often provide the most support, but they also experience greater emotional, financial, and physical burden, despite reporting fewer behavior concerns compared to, for example, children or children-in-law [42]. A fourth limitation is that some interview questions, particularly those related to feeling seen and heard and nursing staff showing emotion, may have been suggestive and potentially nudged participants to endorse these themes, which could have influenced the prominence of these topics in our findings.

### Future research

The results of this study suggest areas for future research. First, additional qualitative research should be conducted on the fundamentals of informal EOL conversations with older people and on the perspectives of family caregivers in relationships other than that of a spouse regarding the fundamentals of EOL communication. Second, the results of this study may be used to develop a theoretical framework for educating nursing staff and for designing and implementing interventions that empower them to take a leading role in EOL communication. However, as interprofessional collaboration is a central aspect of ACP, other professionals involved in EOL communication (e.g., physicians and spiritual caregivers) may have different or enriching views on how to apply these fundamentals. Thus, their views should also be explored to complement the framework.

## Conclusion

This study shows that older people and their family caregivers have few specific expectations about the content and form of EOL conversations and are often not prepared. The most important finding is that older people and their family caregivers prioritize feeling comfortable in natural and humane EOL conversations. They want to be seen, heard, and acknowledged as individuals with backgrounds, values, and needs, not just as patients with a disease. Nursing staff should be aware of the expectations of older people in EOL communication and adjust their approach accordingly. Our findings may facilitate future research, intervention development, and education in EOL communication.

## Supplementary Information

Below is the link to the electronic supplementary material.

Supplementary Material 1

Supplementary Material 2

## Data Availability

The data that support the findings of this study are not publicly available because participants of this study did not give written consent for their data to be shared publicly. However, upon reasonable request, researchers with specific research questions related to this work may contact the first author to discuss potential access to the data for their research purposes.

## Abbreviations

ACP: Advance care planning
EOL: End-of-life
FC: Family caregiver
LISTEN: Learning about the essentIal fundamentalS of EOL communicaTion in palliativE Nursing care
O: Older people
