# Supplementary material for: What matters in end-of-life communication with nursing staff: an interview study among older people and their family caregivers
Source: BMC Nurs. 2025 Sep 26;24:1205. doi: 10.1186/s12912-025-03882-4 (PMC12465498; doi:10.1186/s12912-025-03882-4)
Supplement: Supplementary file 1 — Supplementary Material 1 [file 12912_2025_3882_MOESM1_ESM.docx]

Appendix A: Interview guide

| **Primary questions** | **Probes** |
| --- | --- |
| When did the conversation with the nurse take place? | Location? Duration? Interruptions? |
| **Were you ready for this conversation?** | |
| **Perparation** | |
| **What was the preparation of the conversation like?** | |
| Did you know in advance what the conversation would be about? | What exactly did you know?  Who told you this? |
| Was it the first time the end-of-life was discussed with you? | Were these planned or spontaneous conversations? |
| How was your relationship with the nurse you had the conversation with? | What did you consider important in this?  How long had you known this nurse?  Did you choose the person you interacted with yourself? |
| How did you prepare yourself for the conversation? | Did you get any help with this?  From whom?  How did he/she help you?  *Was your family caregiver present during the conversation?*  *How did your family caregiver prepare for the conversation?*  *Did your family caregiver receive help with this?* |
| Did you feel that the nurse was prepared for the conversation? | How did you notice this?  How did you feel about this? |
| **Conversation** | |
| **What were your feelings about the conversation?** | |
| Did you feel seen? | What made you feel like this? |
| Did you feel heard? | What made you feel like this? |
| What did you like about what the nurse said or did in the conversation? | What happened exactly?  Why did you like that?  Was it a fluent conversation? |
| How did or would you feel about the nurse showing emotion during the conversation? | What made/makes you feel like this? |
| What did you not like about what the nurse said or did in the conversation? | What happened exactly?  Why did you not like that? |
| What topics were discussed during the conversation? | Did you miss any topics?  Why were these topics not discussed? |
| Did you miss anything else during the conversation? | Why did you miss this?  Did the nurse have sufficient knowledge about your culture? |
| **Closing** | |
| **Did the converstion have any follow-up?** | |
| Did the conversation trigger anything? | Did you reflect on the conversation?  Did you talk to anyone about the conversation afterwards? |
